# Combination therapy in hypertension: new recommendations

**Published:** 2010-04

**Authors:** J Aalbers

**Affiliations:** Special Assignments Editor

## Introduction

The recent publication of the American Hypertension Society (ASH) position paper on combination therapy in the treatment of hypertension is essential reading for all physicians and for South African medical aid funders.^1^

As all agree that the goal of antihypertensive therapy is to reduce the risk of vascular events, it is essential that effective and easy-to-use antihypertensives with outcomes data be used early in therapy. Available data from clinical trials and meta-analyses have shown that at least 75% of patients will require combination therapy to achieve contemporary targets. The increasing prevalence of obesity, the metabolic syndrome and type 2 diabetes is likely to increase this percentage even higher, the position paper notes.

When choosing combination therapy, the position paper points to the physician making a deliberate choice as to which combination for which patient. The physician needs to consider efficacy, tolerability and adherence aspects when prescribing combination therapy.

In efficacy terms, rational combination therapy is based on evidence that the combination lowers blood pressure more significantly than its individual components. The blood pressure reduction should be smooth and continuous, meeting pharmacokinetic criteria for once-a-day usage. Dose-dependent effects of the combination should be less than those induced by higher dosage of the monotherapy.

Funders need to take note that co-payment by the medical aid member will reduce compliance and reduce the protective effect of the antihypertensive medication on future vascular events.

The ASH position paper identifies twodrug combinations that meet the three criteria outlined above (a single pill with three or more drugs were not reviewed) and these are regarded as preferred combinations. Others that have less evidence to support efficacy, safety or tolerance are also identified.

## RAAS inhibitor and diuretic

This combination is classified as preferred, whether an ACE inhibitor or an angiotensin receptor blocker (ARB), is used with a low-dose diuretic. Most combinations contain hydrochlorothiazide, but chlorthalidone is also identified as the most-used diuretics in US outcomes trials, although combinations with this diuretic are not currently available.

## RAAS inhibitor and calclum channel blocker

The combination of an ACE inhibitor or ARB with a calcium channel blocker (CCB) results in fully additive blood pressure reduction and improves tolerability. The ACCOMPLISH trial^2^ (Avoiding Cardiovascular events through COMbination therapy in patients living with Systolic Hypertension) showed beneficial cardiovascular outcomes of this ACE inhibitor/CCB combination compared with the ACE inhibitor/diuretic. Most of the patients in this trial were diabetic, with evidence of underlying ischaemic disease. The position paper considers ARB/CCB combinations equivalent to ACE inhibitor/ CCB combinations 

## Renin inhibitor and ARBs

This combination, although without outcome data, has achieved partially additive blood pressure reduction and is well tolerated. In a study of maximum approved doses of valsartan and aliskiren,^3^ a 30% additional blood pressure response was seen compared to monotherapy. The sideeffect profile matched that of placebo.

## CCB and diuretics

This combination also results in partially additive blood pressure reduction and performed well in outcome studies.^4^ It is classified in the position paper as acceptable, perhaps because it does not meet the criteria of reduced side-effect profile of the combination compared to the individual drugs.

## β-blockers and diuretics

The position paper notes that there is evidence, mainly with the first-generation β-blocker, atenolol, that β-blockers are less effective than diuretics, ACE inhibitors, ARBs and CCBs. β-blockers attenuate the RAAS activation that accompanies the use of thiazide diuretics, and their combination results in fully additive blood pressure reduction.

Addition of the diuretic improves the efficacy of β-blockers in black patientsand others with low-renin hypertension.5 These combinations are classed as acceptable with known side effects, such as increased risk of glucose intolerance, fatigue and sexual dysfunction.

## Thiazide diuretics and potassiumsparing diuretics

The use of spironolactone/HCTZ in obese patients is especially noted, as is the fact that the combination should be used only in people with relatively well-preserved kidney function (eGFR > 50 ml/min).

## CCBs and β-blockers

The pharmacological effects of these two drug classes are complementary and result in additive blood-pressure reduction. The combination should be with a dihydropyridine CCB and not a non-dihydropyridine CCB such as verapamil or diltiazen because of their additive effects on heart rate and A–V conduction.

This position paper places ACE inhibitors and ARBs, RAAS inhibitors and β-blockers, and the combination of β-blockers and centrally acting agents in the category of lower efficacy. It concludes that early use of a combination reduces counter-regulatory responses of monotherapy and brings blood pressure to target in a shorter period of time.

## References

[1] Gradman AH, Basile JN, Carter BL, Bakris GL (2010). on behalf of the American Society of Hypertension writing group.. J Am Soc Hypertens.

[2] Jamerson K, Weber MA, Bakris GL, Dahlof B, Pitt B, Shi V (2008). for the ACCOMPLISH trial investigators.. N Engl J Med.

[3] Oparil S, Yarrows SA, Patel S (2007). Lancet.

[4] Julius S, Kjeldsen SE, Weber M, Brunner HR, Ekman S, Hansson L (2004). for the VALUE trial group.. Lancet.

[5] Gradman AH (2008). In: Isso Jl (jun), Black HR, Sica DA, eds.. Hypertension Primer..

